# Structural insights into the antiviral efficacy of AG7404 against human rhinovirus 3C proteases

**DOI:** 10.1107/S2052252525008929

**Published:** 2026-01-01

**Authors:** Juyeon Lee, Hye Lim Lee, Hyojin Kim, Yeji Gil, Sang-Ho Lee, Young-Sik Jung, Jin Soo Shin, Inseong Jo

**Affiliations:** a Infectious Diseases Therapeutic Research Center, Division of Therapeutics and Biotechnology Korea Research Institute of Chemical Technology (KRICT) Daejeon34114 Republic of Korea; b School of Pharmacy Sungkyunkwan University Suwon16419 Republic of Korea; c Department of Chemistry, College of Natural Sciences Seoul National University Seoul08826 Republic of Korea; d Department of Medicinal Chemistry and Pharmacology University of Science and Technology Daejeon34113 Republic of Korea; University of Michigan, USA

**Keywords:** 3C proteases, rhinovirus, antiviral agents, structure determination, drug discovery, X-ray crystallography, antiviral efficacy, binding mechanisms, serotypes

## Abstract

Human rhinoviruses (hRVs) are major causative agents of the common cold and contribute to lower respiratory tract infections. Although extensive research efforts continue to explore potential therapeutic interventions, no clinically approved antiviral treatment currently exists for hRV infections. This study provides the structural basis of hRV 3C protease inhibition in several serotypes by AG7404, a modified rupintrivir derivative. AG7404 showed potent antiviral activity against hRV-B14, hRV-A16 and hRV-A21 with EC_50_ values of 0.108, 0.191 and 0.187 µ*M*, respectively, and directly inhibited purified hRV-B14 3C protease with an IC_50_ of 0.046 µ*M*. The 2.11 Å crystal structure of hRV-B14 3C protease in complex with AG7404 revealed covalent binding to the catalytic Cys146 and occupation of the substrate-binding pockets by the ligand. Comparative structural analyses incorporating ligand-free hRV-B14 3C protease structures as well as rupintrivir-bound 3C protease structures from other hRV serotypes revealed significant conformational variability of the βcII–βdII region. Molecular dynamics simulations of the structural models of hRV-A16 and hRV-A21 3C proteases showed that the binding interactions of AG7404, including critical water-mediated networks, are conserved across serotypes despite sequence variations. These findings offer structural insights into the binding mode of AG7404 and establish a foundation for the rational design of broad-spectrum antivirals targeting hRV 3C proteases.

## Abstract 2

This study presents the crystal structure of human rhinovirus (hRV)-B14 3C protease bound to AG7404, a broad-spectrum antiviral inhibitor, at a resolution of 2.11 Å. Antiviral assays and *in silico* studies provide structural insights into AG7404’s efficacy and binding mechanism against hRV-B14, hRV-A16 and hRV-A21 3C proteases.

## 1. Introduction

Human rhinoviruses (hRVs), members of the *Enterovirus* genus within the *Picornaviridae* family, are a major contributor to the common cold (Simmonds *et al.*, 2020 ▸; Palmenberg *et al.*, 2009 ▸; Jacobs, Lamson *et al.*, 2013 ▸). Also, hRVs contribute to lower respiratory tract infections, severe bronchiolitis and pneumonia, especially in children, often requiring hospitalization and intensive care (Hayden, 2004 ▸; Mohanty *et al.*, 2024 ▸; Jacobs, Soave *et al.*, 2013 ▸; Bastos *et al.*, 2022 ▸). Furthermore, hRVs exacerbate chronic respiratory conditions such as asthma, chronic obstructive pulmonary disease (COPD) and pneumonia in vulnerable populations (Jackson & Gern, 2022 ▸; Gern, 2010 ▸; Jackson, 2010 ▸; Bizot *et al.*, 2021 ▸). In patients with COPD, hRV infections impair alveolar macrophage phagocytosis of bacteria such as *Haemophilus influenzae* and *Streptococcus pneumoniae*, leading to secondary bacterial outgrowth and delayed recovery (Finney *et al.*, 2019 ▸). Immunocompromized patients face higher hospitalization and intensive-care-unit admission rates from hRVs than those observed for pandemic influenza H1N1 (Kraft *et al.*, 2012 ▸). Despite this considerable clinical burden, therapeutic options remain limited, with no approved antiviral therapies for hRV infections (Shahani *et al.*, 2017 ▸; Jacobs, Lamson *et al.*, 2013 ▸).

Initially, hRVs were assigned to ∼100 consecutively numbered serotypes; however, now, more than 160 serotypes have been identified and classified into three species: hRV-A, hRV-B and hRV-C (Simmonds *et al.*, 2020 ▸; Palmenberg *et al.*, 2009 ▸; Ljubin-Sternak & Meštrović, 2023 ▸). The species hRV-A and hRV-C are clinically predominant, associated with severe wheezing and asthma, whereas hRV-B typically causes milder symptoms. The hRV genome includes a single open reading frame that is translated into a polyprotein precursor, sequentially ordered as VP4–VP2–VP3–VP1–2A–2B–2C–3A–3B–3C–3D. The polyprotein is cleaved into mature structural proteins (VP4, VP2, VP3, VP1) and non-structural proteins (2A, 2B, 2C, 3A, 3B, 3C, 3D) via a highly regulated proteolytic cascade. The 3C protease is crucial for polyprotein processing, except at the VP4/VP2 and VP1/2A junctions, generating mature viral components essential for replication (Megremis *et al.*, 2012 ▸; Palmenberg *et al.*, 2010 ▸; Matthews *et al.*, 1994 ▸).

The 3C protease structurally adopts a chymotrypsin-like fold with a well defined active-site cleft, enabling precise substrate recognition and cleavage, making it suitable for selective small-molecule inhibition (Bjorndahl *et al.*, 2007 ▸; Dragovich *et al.*, 1999 ▸). Numerous inhibitors, including covalent and non-covalent, have been developed to target the hRV 3C protease (Liu *et al.*, 2021 ▸; Yuan *et al.*, 2020 ▸; Lockbaum *et al.*, 2021 ▸; Namoto *et al.*, 2018 ▸; De Palma *et al.*, 2008 ▸; Binford *et al.*, 2007 ▸; Kim *et al.*, 2012 ▸; Lacroix *et al.*, 2015 ▸; Papaneophytou, 2024 ▸). The irreversible covalent inhibitor rupintrivir (AG-7088) exhibited broad-spectrum activity against 48 hRV serotypes *in vitro*, with the 50% effective concentration (EC_50_) values ranging from 3 to 81 n*M* (Binford *et al.*, 2005 ▸; Patick *et al.*, 1999 ▸; Matthews *et al.*, 1999 ▸). In phase-II human challenge trials, rupintrivir treatment decreased mean symptom scores by 33%, suggesting potential clinical benefits (Hayden *et al.*, 2003 ▸). However, its clinical development was discontinued owing to limited efficacy and suboptimal pharmacokinetic properties (Li & Peng, 2021 ▸).

AG7404 (also known as Compound 1), a modified rupintrivir derivative, improves pharmacokinetics, demonstrating a threefold-higher oral bioavailability than rupintrivir (Patick *et al.*, 2005 ▸). AG7404 demonstrates broad-spectrum activity against hRVs, inhibiting 35 tested hRV serotypes with EC_50_ values of 14–122 n*M* in cell-based assays. This compound also exhibits potent inhibition against human enteroviruses (hEVs), also members of the *Enterovirus* genus within the *Picornaviridae* family (Tan *et al.*, 2013 ▸; Hu *et al.*, 2020 ▸). Crystallographic studies of AG7404 complexes with 3C proteases from hEV-68 [Protein Data Bank (PDB) ID 8w3m] and hEV-93 (PDB ID 3q3y) (Azzolino *et al.*, 2025 ▸; Costenaro *et al.*, 2011 ▸) provide detailed structural insights into its mechanism. AG7404 also shows antiviral efficacy against SARS-CoV-1 and SARS-CoV-2 by inhibiting 3CL protease, with the structural basis for this interaction thoroughly characterized (Fàbrega-Ferrer *et al.*, 2022 ▸). Despite available structural data for AG7404 complexes with related viral proteases, the structural basis for the interaction between AG7404 and hRV 3C proteases remains unexplored.

This study investigates the structural basis of AG7404-mediated inhibition among rhinovirus serotypes. We demonstrate the antiviral efficacy of AG7404 against hRV-B14, -A16, and -A21 using cell-based and enzymatic assays. Additionally, we provide the crystal structure of the hRV-B14 3C protease–AG7404 complex. For hRV-A16 and -A21 3C proteases, we employed *in silico* modeling and molecular dynamics (MD) simulations to generate binding models for AG7404. Collectively, our findings clarify the conserved binding mode of AG7404 across hRV serotypes and lay the groundwork for structure-guided therapeutic development targeting 3C proteases.

## 2. Materials and methods

### 2.1. Antiviral assay

H1HeLa cells (ATCC CRL-1958) were maintained in Dulbecco’s Modified Eagle’s Medium (DMEM; Corning, USA) supplemented with 5%(*v*/*v*) fetal bovine serum (Hyclone, USA) and 1% antibiotic–antimycotic. Then, hRV B14 (ATCC VR-284), A16 (ATCC VR-283), and A21 (ATCC VR-496) were propagated in the H1HeLa cells and stored at −70°C until further use.

To test the anti-rhinoviral activity, a cytopathic effect (CPE) inhibition assay was performed as previously described (Kumar Biswas *et al.*, 2022 ▸). Briefly, the H1HeLa cells were seeded in 96-well plates (3 × 10^4^ cells per well) and incubated overnight at 37°C in a 5% CO_2_ incubator. The following day, each diluted rhinovirus (100 TCID_50_ ml^−1^) and the threefold serially diluted AG7404 were mixed in equal volumes and incubated at 33°C for 72 h. After incubation, cell viability was measured using the 3-(4,5-dimethylthiazol-2yl)-2,5-diphenyl­tetrazolium bromide (MTT, Sigma) assay, and the absorbance at 540 nm/690 nm was measured using a microplate reader (Synergy H1, Biotek, USA). The 50% effective concentration (EC_50_) and 50% cytotoxicity concentration (CC_50_) were calculated using *GraphPad Prism* 9 (GraphPad Software, San Diego, California, USA, https://www.graphpad.com) via nonlinear regression analysis: log(agonist) versus normalized response – variable slope model.

### 2.2. Protein expression and purification

The gene encoding hRV-B14 3C protease was cloned into the pRSFDuet-1 vector with an N-terminal 10×His-SUMO tag sequence. The plasmids were transformed into *Escherichia coli* BL21(DE3) for protein expression. Transformed cells were cultured in the LB medium containing 50 µg ml^−1^ kanamycin at 37°C until the OD_600_ reached 0.5. Protein expression was induced by adding 0.5 m*M* isopropyl β-d-1-thiogalactopyranoside (IPTG) at 30°C for 4 h. The cells were harvested and stored at −80°C until further purification.

Harvested cells were resuspended in a lysis buffer containing 20 m*M* Tris–HCl (pH 8.0), 150 m*M* NaCl and 2 m*M* 2-mercaptoethanol. The cells were disrupted via sonication, followed by centrifugation at 19 000*g* for 30 min at 4°C to remove insoluble debris. The supernatant was loaded onto Ni–NTA affinity resin pre-equilibrated with lysis buffer. Impurities were removed using a wash buffer containing 20 m*M* Tris–HCl (pH 8.0), 150 m*M* NaCl, 20 m*M* imidazole and 2 m*M* 2-mercaptoethanol. The His-SUMO-3C protease was eluted using an elution buffer containing 20 m*M* Tris–HCl (pH 8.0), 150 m*M* NaCl, 300 m*M* imidazole and 2 m*M* 2-mercapto­ethanol.

The elute was treated with SUMO protease (Ulp1) at 4°C for 16 h to remove the N-terminal His-SUMO tag. After digestion, the mixture was diluted fivefold with 20 m*M* Tris–HCl (pH 8.0) buffer and loaded onto a HiTrap Capto Q ImpRes column (Cytiva, USA). The His-SUMO tag was retained on the resin and the cleaved 3C protease was passed through. The flow-through fraction containing the target protein was subjected to size-exclusion chromatography (SEC) using a HiLoad Superdex 200 pg 16/600 column (Cytiva, USA) pre-equilibrated with SEC buffer composed of 20 m*M* Tris–HCl (pH 8.0), 150 m*M* NaCl and 2 m*M* tris(2-carboxyethyl)phosphine (TCEP). The purified 3C protease was concentrated at 10 mg ml^−1^ and stored at −80°C.

### 2.3. Enzymatic assay

The inhibitory activity of AG7404 against hRV-B14 3C protease was determined using a colorimetric assay performed at 30°C in a reaction buffer containing 25 m*M* HEPES (pH 7.5), 150 m*M* NaCl, 1 m*M* EDTA and 1 m*M* TCEP. The 3C protease and inhibitors were prepared in a series of concentrations using DMSO as the solvent and pre-incubated in the reaction buffer. The reaction was initiated by adding 250 µ*M* of the colorimetric peptide substrate EALFQ-pNA, synthesized by LugenSci, Inc. (Republic of Korea). This substrate was designed based on a previously reported study (Wang *et al.*, 1997 ▸). Cleavage of the EALFQ-pNA peptide substrate by the 3C protease released *p*-nitroaniline (pNA), which was quantified by measuring absorbance at 405 nm using a microplate reader (Spark 10M, Tecan, Switzerland) in kinetic mode. The IC_50_ values were determined using the initial reaction velocities.

### 2.4. Crystallization and X-ray data collection

The purified hRV 3C protease was incubated with the covalent inhibitor AG7404 at a molar ratio of 1:2, resulting in final concentrations of 0.5 m*M* protein and 1 m*M* inhibitor. Crystallization drops were prepared by mixing 1 µl of the protein–inhibitor complex solution with 1 µl of precipitant solution and equilibrating against a 500 µl reservoir. Crystallization screening was performed using the vapor diffusion method and the best crystals were grown at 14°C in a precipitant solution containing 0.1 *M* Tris–HCl (pH 8.0), 0.8 *M* LiCl and 36%(*w*/*v*) PEG 4000. Crystals were briefly soaked in cryoprotectant (reservoir solution with 25% glycerol) and flash cooled in liquid nitrogen for data collection. X-ray diffraction data were collected at the Pohang Accelerator Laboratory Beamline 5C SB II at a wavelength of 1.00003 Å (Park *et al.*, 2017 ▸).

### 2.5. Structure determination

The diffraction data were indexed and scaled using *XDS* (Kabsch, 2010 ▸), with truncation based on the criteria of *I*/σ(*I*) > 2.0, CC_1/2_ > 70% and completeness > 70% in the highest-resolution shell. The phase problem was solved via molecular replacement using *Phaser-MR*, as implemented in *Phenix* (McCoy *et al.*, 2007 ▸; Liebschner *et al.*, 2019 ▸). Model building and iterative refinement were conducted using *Coot* and *phenix.refine* (Afonine *et al.*, 2012 ▸; Liebschner *et al.*, 2019 ▸). The final structure was visualized and analyzed using *PyMOL* (Schrodinger LLC, 2015 ▸).

### 2.6. Modeling hRV-A16 and -A21 3C proteases with AG7404

To investigate the AG7404 binding mode in hRV-A16 and -A21 3C proteases, the monomeric structures of the proteases were predicted using *AlphaFold3* (Abramson *et al.*, 2024 ▸). The hRV-A16 3C protease (NCBI Accession Number: AAA69862.1) and hRV-A21 3C protease (NCBI Accession Number: AKO83942.1) sequences were obtained from the NCBI database. The generated structures were evaluated for quality using per-residue confidence score (pLDDT) and predicted aligned error (PAE) scores.

To create AG7404 models covalently bound to the predicted structures, the hRV-B14 3C crystal structure was used as a template for docking. Subsequently, we prepared input files for MD simulations using the *CHARMM-GUI Solution Builder* (Lee *et al.*, 2016 ▸). Each complex structure was placed in a cubic simulation box, solvated with TIP3P water molecules and neutralized with 150 m*M* KCl. The CHARMM36m force field was applied to the system (Huang *et al.*, 2017 ▸).

Energy minimization, equilibration and MD simulations were performed using *GROMACS* (Abraham *et al.*, 2015 ▸). Energy minimization used the steepest descent method, followed by NVT equilibration for 125 ps at 303.15 K with Nosé–Hoover temperature coupling (Nosé, 2002 ▸; Hoover, 1985 ▸). A 100 ns MD simulation was subsequently conducted under the NPT ensemble at 1 atm and 303.15 K with the Parrinello–Rahman barostat (Parrinello & Rahman, 1981 ▸). A time step of 2 fs was used, and the resulting trajectories were analyzed to assess complex stability using *GROMACS* analysis tools.

## 3. Results and discussion

### 3.1. Inhibition of hRV-B14 3C protease by AG7404

**Figure 1:**
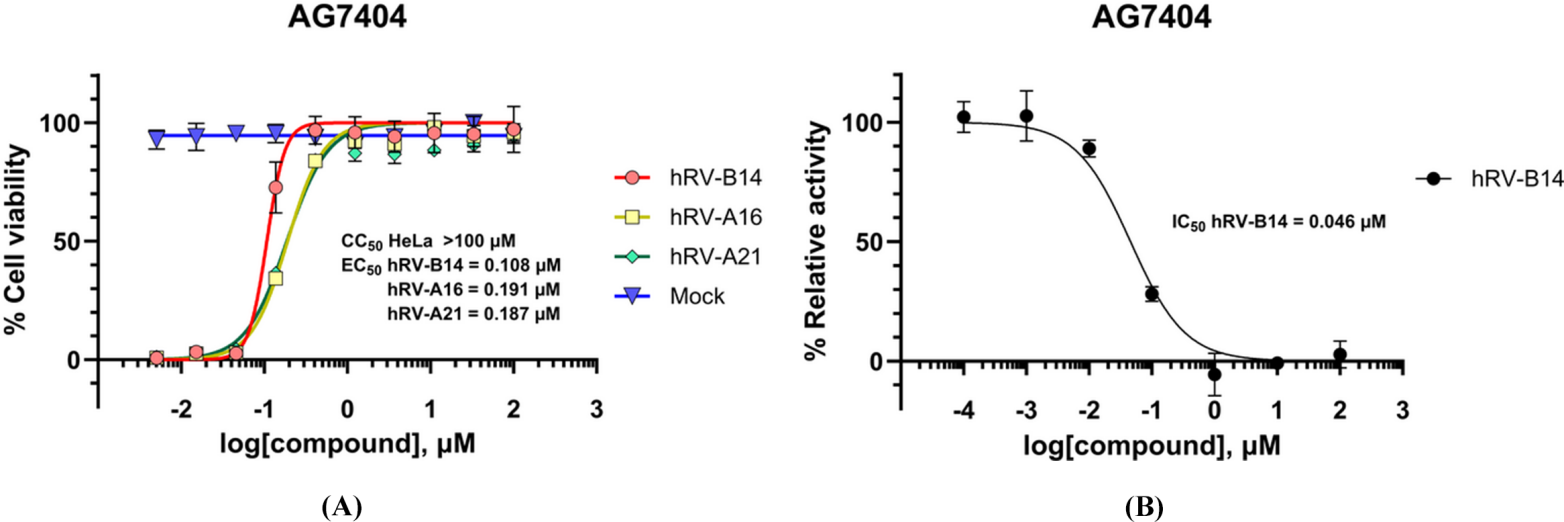
Anti-rhinoviral efficacy and enzymatic inhibition of AG7404. (*A*) Dose-response curve analysis of AG7404 against hRV-B14, -A16 and -A21. HeLa cells were mock-infected (blue triangle) or infected with hRV-B14 (red circle), hRV-A16 (yellow square) and hRV-A21 (green rhombus) with various concentrations of AG7404. After 72 h, cell viability was measured using the MTT assay. Data represent means (±SD) from at least two independent experiments performed in duplicate. (*B*) Inhibitory activity of AG7404 against purified hRV-B14 3C protease. The enzymatic assay was performed using a colorimetric peptide substrate (EALFQ-pNA), and the release of pNA was measured at 405 nm to calculate the IC_50_ value. Data represent means (±SD) from triplicate experiments.

The antiviral efficacy of AG7404 against three rhinovirus serotypes, hRV-B14, hRV-A16 and hRV-A21, was evaluated using the CPE assay. H1HeLa cells were infected with each virus and treated with threefold serial dilutions of AG7404 for 72 h. AG7404 exhibited potent antiviral activity with EC_50_ values of 0.108 µ*M*, 0.191 µ*M* and 0.187 µ*M* for hRV-B14, hRV-A16 and hRV-A21, respectively, with no cytotoxicity observed at the tested concentrations (CC_50_ > 100 µ*M*) [Fig. 1 ▸(*A*)].

To investigate the molecular basis of inhibition, we selected hRV-B14 3C protease for further structural studies and directly assessed the inhibitory potency of AG7404 against purified hRV-B14 3C protease using an enzymatic assay. This assay employed a colorimetric peptide substrate reported in a previous study (Wang *et al.*, 1997 ▸). The colorimetric peptide contains the EALFQ sequence, corresponding to the viral 3C protease recognition motif, with a para-nitroaniline (pNA) reporter group at the C-terminus. Proteolytic cleavage of this substrate releases the pNA moiety, enabling quantitative enzyme activity measurements via absorbance at 405 nm. AG7404 strongly inhibited hRV-B14 3C protease with an IC_50_ of 0.046 µ*M*, consistent with the antiviral potency observed in the cellular assay and confirming direct 3C protease activity inhibition [Fig. 1 ▸(*B*)].

### 3.2. The overall structure of hRV-B14 3C protease with AG7404 inhibitor

To understand the structural basis of AG7404 inhibition, the crystal structure of hRV-B14 3C protease in complex with AG7404 was determined at a resolution of 2.11 Å. Crystals were obtained by co-crystallizing the purified protease with AG7404 at a 1:2 molar ratio. They belonged to the *P*2_1_ space group with unit-cell dimensions of *a* = 32.88, *b* = 148.66, *c* = 67.41 Å and β = 92.62°. The complex structure was solved using molecular replacement, containing four protomers in the crystallographic asymmetric unit, with refined *R*_work_ and *R*_free_ values of 22.5% and 26.7%, respectively. The statistics of X-ray diffraction data and refinement are summarized in Table 1 ▸.

**Figure 2:**
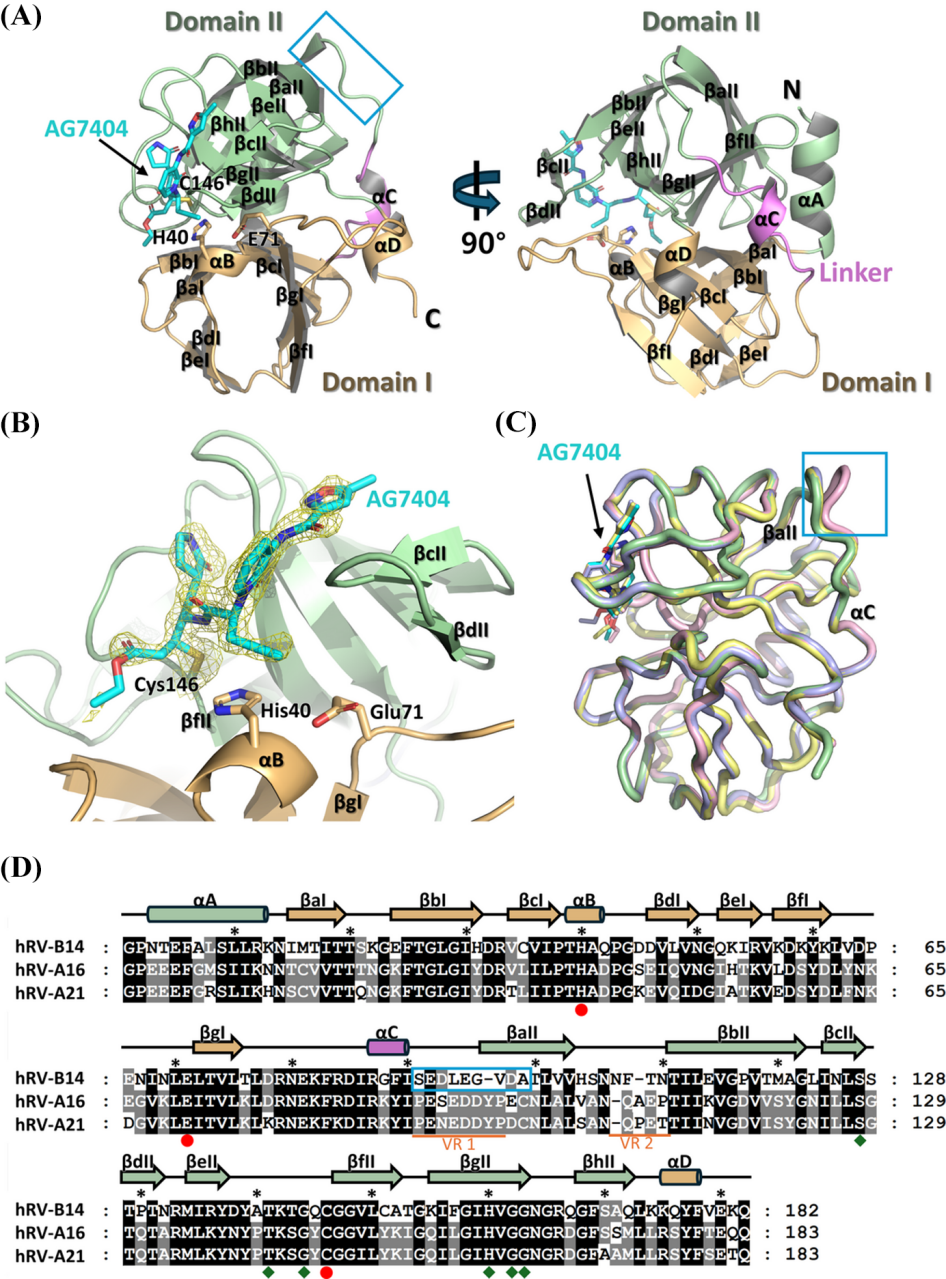
Overall structure of the hRV-B14 3C protease bound to the AG7404 inhibitor. (*A*) Orthogonal views of the hRV-B14 3C protease–AG7404 complex structure with domain I in brown, domain II in green, and the linker region connecting the two domains in violet. The catalytic triad residues (Cys146, His40 and Glu71) are labeled and secondary structural elements (α-helices and β-strands) are annotated. AG7404 is depicted as a cyan stick model positioned within the active site. The blue box indicates a region showing minor conformational differences among protomers in the asymmetric unit. (*B*) Close-up view of the active site showing AG7404 covalently bonded to Cys146. A polder map was generated using *phenix.polder* after omitting Cys146 and AG7404, with the resulting electron density shown as a yellow mesh contoured at 3.0σ. The residues of the catalytic triad are in stick representation. (*C*) The superposition of the four protomers in the asymmetric unit, presenting structural conservation and minor conformational variability in the loop between αC and βaII (blue box). The AG7404 molecules are shown as stick models bound at identical positions across all protomers. (*D*) Sequence alignment of hRV-B14, hRV-A16 and hRV-A21 3C proteases with secondary structural annotations derived from the hRV-B14 structure. Conserved residues are shaded in gray and black. The flexible region in the superposition of the four protomers is indicated by a blue box. The variable regions (VR1: residues 91–97; VR2: residues 107–110) derived from sequence alignments between hRV serotypes are underlined in orange. Catalytic residues (red circles) and AG7404-interacting residues (green diamonds) are highlighted.

The hRV-B14 3C protease adopts a chymotrypsin-like fold comprising two β-barrel domains (domains I and II), connected by a linker containing an αC helix. Domain I is composed of seven β-strands (βaI–βgI), an αB helix between βcI and βdI, and a C-terminal αD helix. Domain II consists of eight β-strands (βaII–βhII) and an N-terminal αA helix [Figs. 2 ▸(*A*) and 2 ▸(*D*)]. The catalytic triad of hRV 3C protease consists of Cys146 in domain II, and His40 and Glu71 residues in domain I. A continuous electron-density map extending from the sulfur atom of Cys146 was interpreted as AG7404 within the catalytic site. When we evaluated ligand occupancy using a polder map with AG7404 and Cys146 omitted from the model, the resulting electron density clearly supported the correct placement of the covalent inhibitor in the active site [Fig. 2 ▸(*B*)]. Structural alignment of the four protomers revealed high similarity, with root mean square deviation (RMSD) values of 0.20–0.22 Å across 160 aligned Cα atoms [Fig. 2 ▸(*C*)]. The only minor conformational difference was observed in the loop between αC and βaII of chain C [indicated by blue boxes in Figs. 2 ▸(*A*), 2 ▸(*C*) and 2 ▸(*D*)]. AG7404 molecules were observed at the active site of each protomer [Fig. 2 ▸(*C*) and Fig. S1 of the supporting information].

### 3.3. Detailed interactions of AG7404 with the hRV-B14 3C protease active site

**Figure 3:**
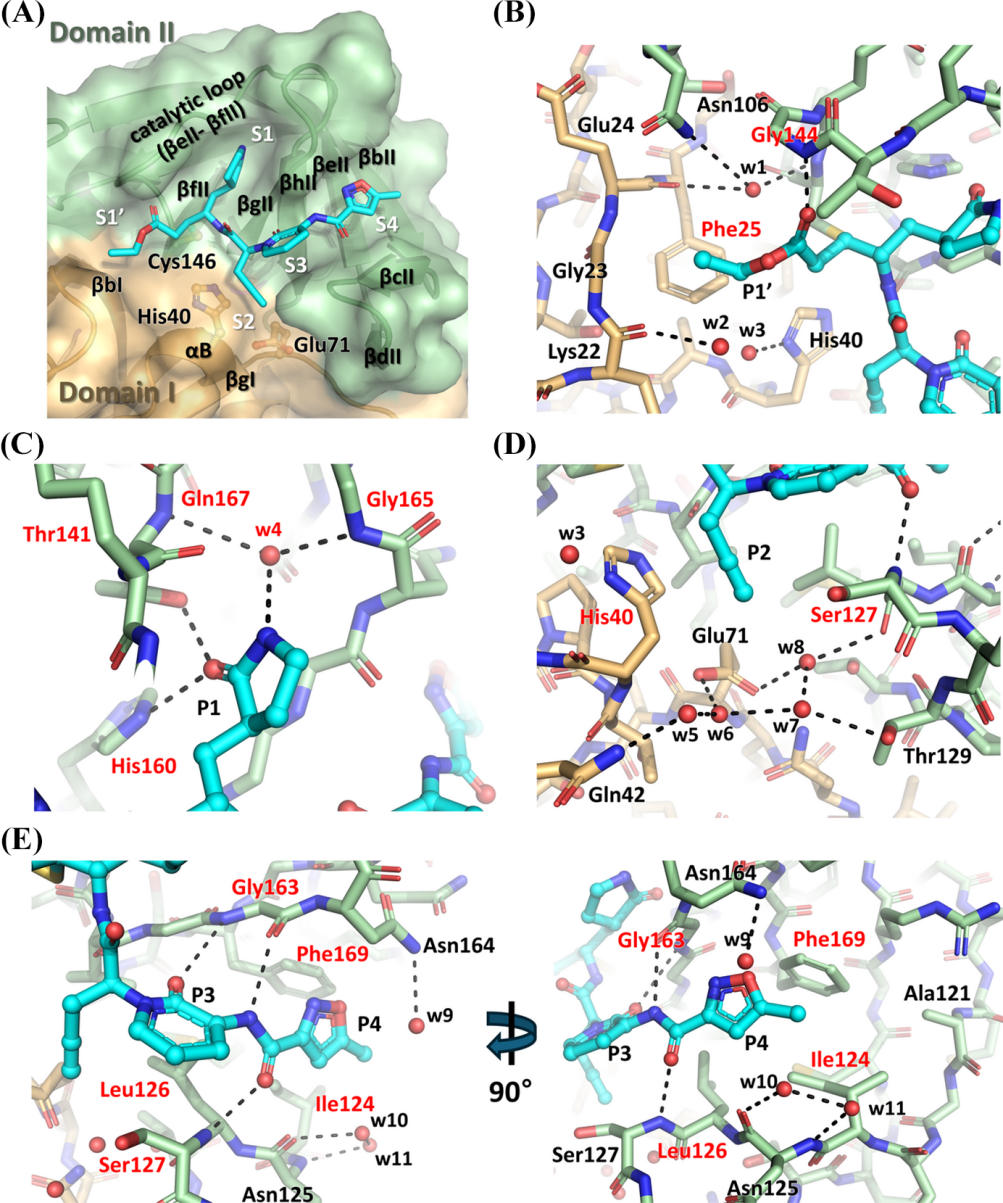
Detailed interactions of AG7404 within the hRV-B14 3C protease. (*A*) Surface representation of the substrate-binding site of hRV-B14 3C protease displays the S1, S2, S3 and S4 pockets. AG7404 is represented as a cyan stick model, with catalytic residues (Cys146, His40 and Glu71) labeled. Secondary-structural elements forming the substrate-binding pockets (S1′, S1, S2, S3 and S4) are annotated. (*B*), (*C*), (*D*), (*E*) Enlarged views of the P1′, P1, P2 and P3–P4 moieties are presented, respectively. Residues and water molecules that directly interact with AG7404 (cyan) are labeled in red. Residues and water molecules participating in the hydrogen-bond network via water molecules, without interacting with AG7404, are marked in black.

AG7404 occupies the substrate-binding cleft of the hRV-B14 3C protease via a well defined binding mode, with its P1′, P1, P2 and P3–P4 moieties positioned in the corresponding substrate recognition pockets S1′, S1, S2 and S3–S4, respectively [Fig. 3 ▸(*A*)]. The superposition of these protomers exhibited minimal structural variability in the P1 to P4 moieties of AG7404. In contrast, the P1′ moiety displayed significant pose variability (Fig. S1).

The S1′ pocket, formed by βbI and the catalytic loop between βeII and βfII, accommodates the P1′ ethyl ester moiety of AG7404. The ester group was stabilized by a hydrogen bond with the backbone amide group of Gly144. However, the ethyl group forms weak interactions with the side chain of Phe25, resulting in a variable orientation of the ethyl moiety of P1′ [Fig. 3 ▸(*B*) and S1]. Nearby, three water molecules are bound: w1 interacts with the side chain of Asn106, and the backbones of Glu24 and Gly144; w2 binds to the backbone of Lys22; and w3 associates with the side chain of His40 [Fig. 3 ▸(*B*)]. These water positions represent potential targets for optimization via the incorporation of hydrogen-bond donors or acceptors at the P1′ position.

The S1 pocket, formed by βgII and the catalytic loop connecting βeII and βfII, interacts via the P1 γ-lactam moiety. The carbonyl group of the γ-lactam moiety at P1 forms direct hydrogen bonds with the His160 and Thr141 residues. Additionally, the γ-lactam amide group participates in a water-mediated hydrogen-bonding network involving w4, bridging to the backbones of Thr141 and Gly165 [Fig. 3 ▸(*C*)]. Similar water-mediated interactions have been observed in hEV-68 3C protease structures and partially in the hEV-93 3C protease with half occupancy (Tan *et al.*, 2013 ▸; Costenaro *et al.*, 2011 ▸; Azzolino *et al.*, 2025 ▸). Therefore, optimizing the P1 moiety to occupy the position of w4 and interact with the backbones of Thr141 and Gly165 could enhance binding affinity. However, as the γ-lactam amide in the 3CL protease of SARS-CoV-1 and -2 directly interacts with the backbone carbonyl and glutamate side chain of the protease (Fàbrega-Ferrer *et al.*, 2022 ▸), this approach is likely limited to developing antiviral agents specifically targeting enterovirus 3C proteases, including hRV and hEV.

The S2 pocket, formed by αB and the loop between βcII–βdII, accommodates the P2 ethynyl group. This linear moiety extends into a narrow hydrophobic cavity, interacting with the Cβ of His40 and Cδ of Ser127 [Fig. 3 ▸(*D*)]. Adjacent to the P2 moiety, a hydrogen-bonding network is observed, involving water molecules (w5–w8) that interact with the side chains of Glu71, Gln42 and Thr129, and the backbone carbonyl group of Leu126 [Fig. 3 ▸(*D*)]. These interactions presented opportunities for optimizing the P2 moiety to enhance binding specificity and affinity.

The S3–S4 pocket, formed by βcII, βbII, βeII, βhII and βgII strands, interacts with the P3–P4 moieties of AG7404. The carbonyl and amide groups of the P3 moiety form hydrogen bonds with the backbone amide and carbonyl groups of Gly163, respectively, mimicking the backbone interactions in β-sheets. The carbonyl group of the P4 moiety establishes a hydrogen bond with the backbone amide of Ser127. Furthermore, the side chains of the Leu126, Ile124 and Phe169 residues participate in hydrophobic interactions with the moieties [Fig. 3 ▸(*E*)]. Adjacent to the P4 moiety, three water molecules (w9–w11) bound to the Asn164 side chain and the Asn125 backbone, alongside a hydrophobic patch formed by the Ala121, Ile124 and Phe169 residues, are notable targets for optimizing the P4 moiety [Fig. 3 ▸(*E*)].

### 3.4. Conformational changes of the βcII–βdII region driven by inhibitor binding

**Figure 4:**
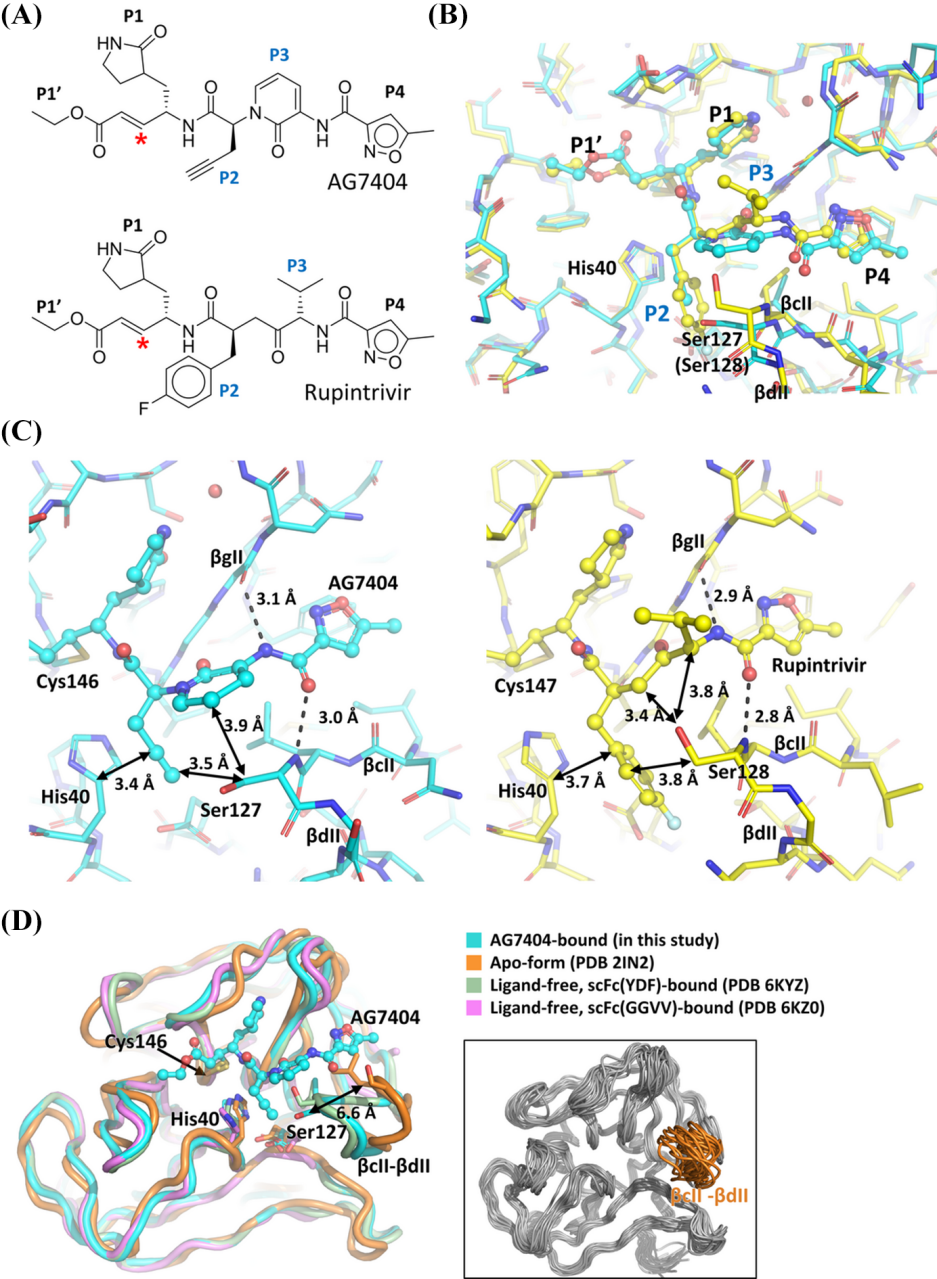
Comparative structural analysis of inhibitor-bound and ligand-free 3C protease. (*A*) Chemical structures of AG7404 (top) and rupintrivir (bottom), highlighting distinct P2 and P3 substituents (blue) and the covalent warhead (red asterisk). (*B*) Superposition of active-site regions from AG7404-bound hRV-B14 3C protease (cyan) and rupintrivir-bound hRV-A2 3C protease (yellow; PDB ID 1cqq). Key features (βcII, βdII, Ser127/Ser128, His40) and inhibitor positions are indicated. (*C*) Close-up view of the βcII–βdII region in AG7404-bound hRV-B14 3C protease (cyan, left) and rupintrivir-bound hRV-A2 3C protease (yellow, right). Representative distances are annotated. (*D*) Overlay of AG7404-bound (cyan), apo-form NMR (orange; PDB ID 2in2), ligand-free scFc(YDF)-bound (green; PDB ID 6kyz) and ligand-free scFc(GGVV)-bound (pink; PDB ID 6kz0) hRV-B14 3C protease structures, illustrating the diversity and disorder of the βcII–βdII region. In the right-hand box, an NMR ensemble shows the conformational variability of the βcII–βdII loop (orange).

AG7404 is a structural derivative of rupintrivir, with substitutions only at the P2 and P3 positions; specifically, AG7404 features an ethynyl substituent at P2 and a 2-pyridon-1,3-diyl moiety at P3, while rupintrivir has a 1-fluorophenyl group at P2 and a valine-derived residue at P3 [Fig. 4 ▸(*A*)]. To analyze the structural consequences of these modifications, we superimposed the hRV-B14 3C protease in complex with AG7404 with the structures of hRV-C15 and -A2 3C proteases bound to rupintrivir (PDB IDs 6ku8 and 1cqq) (Yuan *et al.*, 2020 ▸; Matthews *et al.*, 1999 ▸). Structural superposition of the three complexes revealed that the P1 moiety consistently adopted the same binding pose, including a water-mediated hydrogen bond at the lactam nitrogen of the P1 group in the structure of the hRV-C15 3C protease [Figs. 3 ▸(*C*) and S2]. The P1′ group exhibited variable orientations among the complexes, consistent with the variation observed between individual protomers in the AG7404-bound crystal structure (Figs. S1 and S2). Although the P4 moiety is also common to both inhibitors, its position was slightly shifted between the complexes [Fig. 4 ▸(*B*)]. This positional shift appeared to result from the rigid planar geometry of the P3 2-pyridon-1,3-diyl moiety in AG7404, which constrained the three-dimensional conformation of AG7404 and subsequently affected the positioning of the βcII–βdII region [Figs. 4 ▸(*A*) and 4 ▸(*B*)].

The conformational state of the βcII–βdII region was primarily influenced by the position of Ser127 (or Ser128), which reflected the steric profile of the bound inhibitor. The backbone amine of Ser127 formed a hydrogen bond with the carbonyl group connecting the P3 and P4 moieties (3.0 Å for AG7404; 2.8 Å for rupintrivir). The side chain of Ser127 (or Ser128) was located within the steric environment defined by the P2 and P3 groups of each inhibitor. In the AG7404 complex, the β-carbon of Ser127 was 3.5 Å from the P2 ethynyl group and 3.9 Å from the P3 2-pyridon-1,3-diyl moiety. For rupintrivir, these distances of Ser128 were 3.8 Å from P2 1-fluorophenylalanine and 3.4–3.8 Å from the P3 backbone. This arrangement showed that Ser127 (or Ser128) adjusted to accommodate each ligand, and when AG7404 was bound, the greater bulk of the P3 group resulted in a more distant positioning of the βcII–βdII region [Fig. 4 ▸(*C*)].

To further explore the intrinsic conformational landscape of this region, we compared the AG7404-bound structure with available ligand-free structures of hRV-B14 3C protease. A true apo form is only available as an NMR ensemble (PDB ID 2in2; Bjorndahl *et al.*, 2007 ▸), whereas all reported ligand-free crystal structures of hRV-B14 3C protease are in complex with inhibitory single-chain antibody fragments (scFc YDF and scFc GGVV; PDB IDs 6kyz and 6kz0) (Meng *et al.*, 2020 ▸). In the scFv YDF-bound structure, the βcII–βdII loop adopted a well defined conformation that closely resembled the inhibitor-bound state, which is likely attributable to crystal packing since Ser128 of the βcII–βdII region forms a direct interaction with the scFv YDF. In contrast, in the scFv GGVV-bound structure, the βcII–βdII segment was disordered [Fig. 4 ▸(*D*); pink], and in one of the NMR ensembles, this region was displaced by as much as 6.6 Å at Ser127 [Fig. 4 ▸(*D*); orange]. Analysis across the NMR ensemble highlighted the intrinsic flexibility of the βcII–βdII region, which is consistent with the previous report that inhibitor binding leads to a pronounced reduction in RMSD within this region (Bjorndahl *et al.*, 2007 ▸). These findings underscore that the βcII–βdII region possesses considerable conformational plasticity, while inhibitor engagement induces a well ordered conformation in this region.

### 3.5. Conserved AG7404 binding interactions in hRV-A16 and -A21 3C proteases

**Figure 5:**
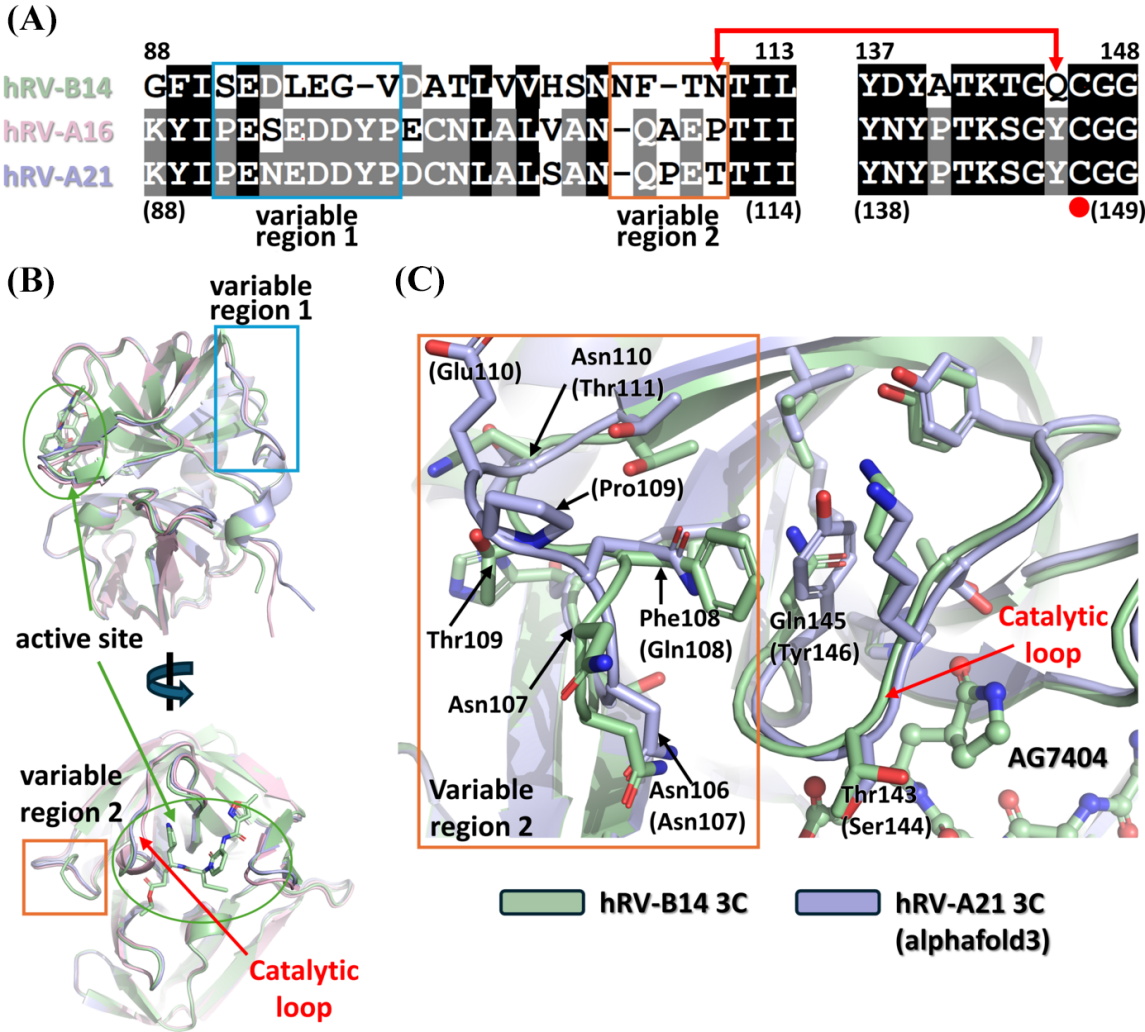
Sequence and structural comparison of hRV-B14, hRV-A16 and hRV-A21 3C proteases. (*A*) Sequence alignment of hRV-B14, hRV-A16 and hRV-A21 3C proteases. Conserved residues are shaded in gray and black, and variable regions (VR1: residues 91–97; VR2: residues 97–110) are highlighted with blue boxes. Red arrows indicate interacting residues. (*B*) Structural superposition of hRV-B14 (green), hRV-A16 (pink; *AlphaFold*-predicted model) and hRV-A21 (light blue; *AlphaFold*-predicted model) 3C proteases. Variable region 1 (blue box) is positioned opposite the active site (green circle) and variable region 2 (orange box) is located near the substrate-binding cleft. (*C*) A close-up view of variable region 2 (orange box) and the catalytic loop (red arrow) of hRV-B14 (green) and hRV-A21 (light blue) 3C proteases. The key residues of hRV-B14 3C protease are denoted without parentheses, whereas hRV-A21 3C protease residues are shown in parentheses.

Monomeric models of hRV-A16 and hRV-A21 3C proteases, which showed antiviral efficacy in the CPE assay, were predicted using *AlphaFold3* (Abramson *et al.*, 2024 ▸). The PAE plot confirmed the reliability of these models (Fig. S3). Structural comparison with the hRV-B14 3C crystal structure revealed high conservation, with RMSD values of only 0.42 Å (144 Cα atoms) and 0.41 Å (152 Cα atoms) for hRV-A16 and hRV-A21 3C proteases, respectively. However, notable sequential and structural differences were observed in two distinct regions: residues 91–97 (variable region 1) and residues 107–110 (variable region 2) [Figs. 5 ▸(*A*) and 5 ▸(*B*)].

Variable region 1, located between domain II and the linker helix (αC), is positioned opposite the active site [Figs. 2 ▸(*A*), 2 ▸(*D*) and 5 ▸(*B*)] and showed conformational variability even among the four protomers in our hRV-B14 3C crystal structure, suggesting minimal impact on enzymatic activity or inhibitor binding [Fig. 5 ▸(*B*)]. In contrast, variable region 2 may influence active-site composition, warranting further analysis [Fig. 5 ▸(*B*)]. Specifically, the Phe108 residue in hRV-B14 3C, which interacts with the catalytic loop (between βeII–βfII) critical for S1 binding-site configuration, was substituted with Gln in -A16 and -A21 3C. Similarly, the Gln145 residue in hRV-B14 3C, which interacts with the Phe108 residue, was replaced by Tyr in -A16 and -A21 3C. With the dual substitution of these two residues, the structural conservation maintained similar residue interactions in this region. Notably, the positions of Asn106 (Asn107 of hRV-A16 and hRV-A21), directed toward the S1 binding site, are conserved across all three serotypes [Fig. 5 ▸(*C*)].

To further assess the reliability of our structural models specifically in inhibitor-binding regions, we analyzed the per-residue measure of local confidence (pLDDT) of *AlphaFold*-predicted structures. This analysis revealed lower confidence in three key regions: residues (i) 125–130, (ii) 141–144 and (iii) 164–167 (Fig. S4). Notably, these regions correspond to those that undergo substantial conformational changes upon AG7404 binding in the hRV-B14 3C crystal structure [Figs. S4 and 4 ▸(*A*)]. Based on these findings, MD simulations were deemed essential for accurately assessing AG7404 interactions across serotypes.

**Figure 6:**
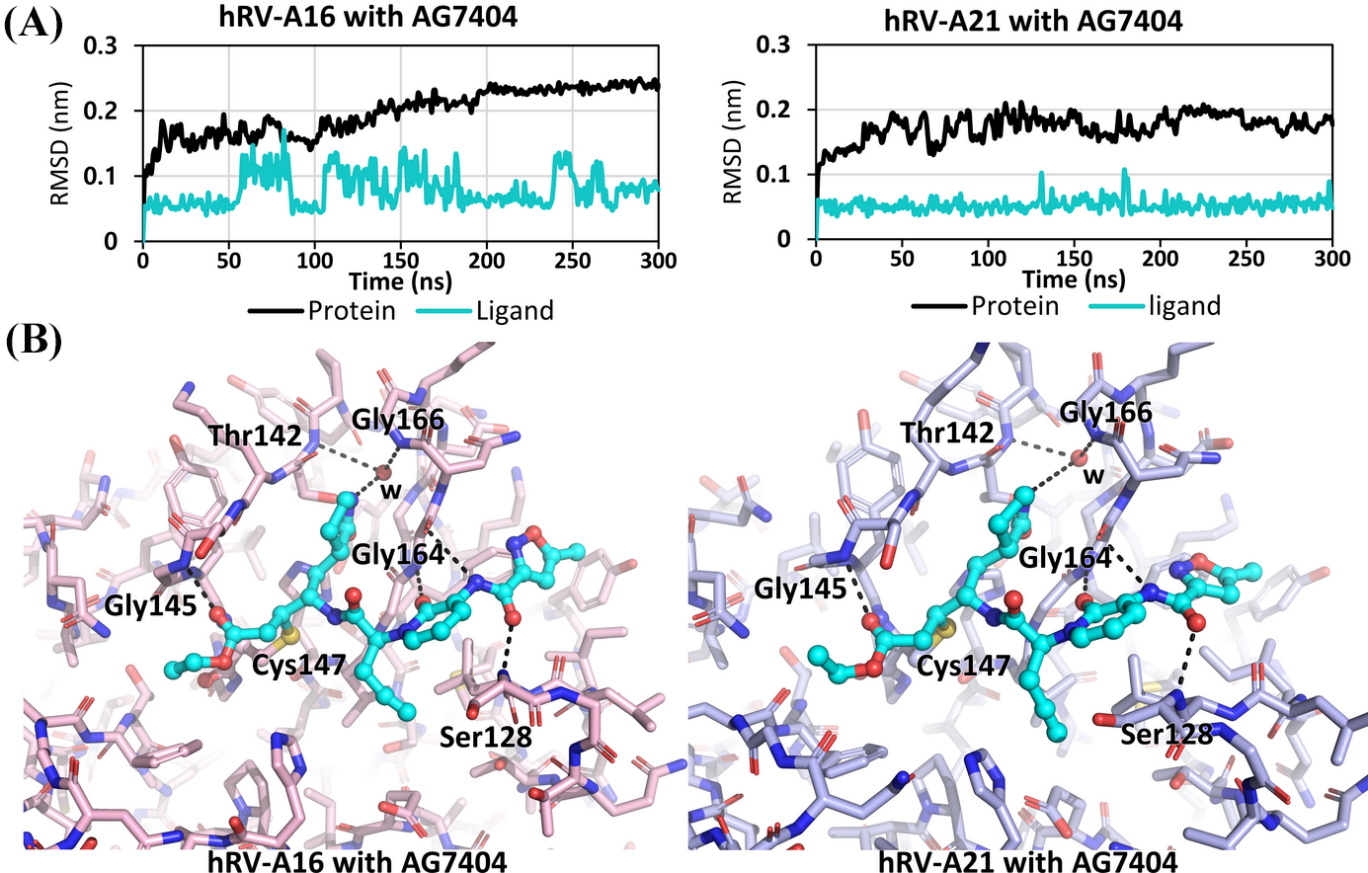
MD simulations of AG7404-bound hRV-A16 and hRV-A21 3C proteases. (*A*) RMSD plots of AG7404-bound hRV-A16 (left) and hRV-A21 (right) 3C proteases for 300 ns of MD simulations. The protein RMSD is depicted in black and the ligand RMSD is displayed in cyan. (*B*) AG7404-bound hRV-A16 (left) and hRV-A21 (right) 3C protease structures at the end of the 300 ns MD simulation. AG7404 molecules are depicted as a cyan ball-and-stick model. Key residues and the water molecules (w) interacting with AG7404 are labeled.

Models representing AG7404 covalently bound to *AlphaFold*-predicted hRV-A16 and hRV-A21 3C proteases were constructed. Energy minimization, equilibrium, and subsequent 300 ns MD simulations using *GROMACS* showed that protein and ligand (AG7404) RMSD values increased and stabilized after 300 ns [Fig. 6 ▸(*A*)]. Structural analysis of the equilibrated complexes revealed that both hRV-A16 and hRV-A21 3C proteases maintained binding interactions with AG7404, consistent with the results observed in our hRV-B14 3C crystal structure [Fig. 6 ▸(*B*)]. Notably, despite using Monte Carlo solvation, key water molecules mediating interactions with AG7404’s P1 moiety exhibited spatial arrangements similar to those in the crystal structure of hRV-B14 3C [Fig. 6 ▸(*B*)]. These findings suggested that the direct and water-mediated interactions between AG7404 and 3C proteases are conserved across hRV serotypes. Simulation-derived models provide a crucial foundation for rational inhibitor design targeting diverse hRV serotypes.

## 4. Conclusions

This study elucidates the structural and functional basis of AG7404-mediated inhibition of hRV 3C proteases, providing a foundation for rational design of antivirals targeting hRV-3C proteases. AG7404 exhibited potent antiviral activity against hRV-B14, hRV-A16 and hRV-A21 with EC_50_ values of 0.108 µ*M*, 0.191 µ*M* and 0.187 µ*M*, respectively. The compound showed low cytotoxicity (CC_50_ > 100 µ*M*), resulting in selectivity indices of 926, 524 and 535 for each serotype, respectively. Direct enzymatic inhibition of purified hRV-B14 3C protease (IC_50_ = 0.046 µ*M*) confirmed its mechanism of action and validated 3C protease as a viable antiviral target.

The crystal structure of the hRV-B14 3C protease–AG7404 complex revealed AG7404 covalently binding to the catalytic Cys146 residue, with its P1′–P4 moieties occupying the substrate-binding pockets. Key interactions included hydrogen bonds between the P1 γ-lactam moiety and His160/Thr141 residues, hydrophobic interactions between the P2 ethynyl group and His40/Ser127, and β-sheet-like hydrogen-bonding patterns with Gly163. Notably, a water-mediated hydrogen-bonding network at the P1 moiety bridges to the backbones of Thr141 and Gly165. This feature is consistent with similar water-mediated interactions observed in other enterovirus (EV) 3C protease structures, such as those for EV-68 and EV-93.

Structural comparison with ligand-free hRV-B14 3C protease and rupintrivir-bound 3C protease from other hRV serotypes revealed that the βcII–βdII region displays significant conformational variability. This highlights the importance of loop plasticity in inhibitor binding and structure-based drug design for hRV 3C protease. MD simulations of the 3C protease–AG7404 complex model of hRV-A16 and -A21 confirmed that the binding mode of AG7404, particularly the water-mediated network near the P1 moiety, is conserved despite sequence variations in variable regions 1 (residues 91–97) and 2 (residues 107–110).

These findings provide a structural blueprint for optimizing AG7404 derivatives. The water-mediated hydrogen-bonding networks around AG7404 present opportunities for structure-guided modifications to enhance binding affinity and specificity. Furthermore, the conformational adaptability of the active site highlights the importance of considering protein flexibility in computational drug design. This study establishes a foundation for developing improved antiviral therapeutics against rhinoviruses.

## Supplementary Material

Figs. S1 to S4. DOI: 10.1107/S2052252525008929/jt5081sup1.pdf

PDB reference: hRV-B14 3C protease in complex with AG7404, 9lgp

## Figures and Tables

**Table 1:** Statistics of X-ray diffraction data and structure refinement Values in parentheses refer to the highest-resolution shell.

Data collection	
Diffraction source	PAL/PLS beamline 5C
Wavelength (Å)	1.00003
Temperature (K)	100
Space group	*P*2_1_
*a*, *b*, *c* (Å)	32.88, 148.66, 67.41
α, β, γ (°)	90, 92.62, 90
Mosaicity (°)	0.34
Resolution range (Å)	29.47–2.11 (2.18–2.11)
Total No. of reflections	255494 (23739)
No. of unique reflections	36418 (3385)
Completeness (%)	98.2 (96.8)
Redundancy	7.0 (7.0)
〈*I*/σ(*I*)〉	11.6 (4.5)
*R*_meas_ †	0.166 (0.685)
*R*_p.i.m._ ‡	0.062 (0.2546)
CC_1/2_ (%)	99.3 (88.3)
CC (%)	99.8 (96.8)

Refinement	
Resolution range (Å)	29.47–2.11
Completeness (%)	98.2
No. of reflections, working set	36414
No. of reflections, test set	1820
*R*_work_ (%)	22.5
*R*_free_ (%)	26.7
No. of non-H atoms	
protein	5488
ligand	152
water	691
total	6331
RMSD	
bonds (Å)	0.001
angles (°)	0.68
Average *B* factors (Å^2^)	
protein	14.0
ligand	11.6
water	14.7
Ramachandran plot	
favored (%)	94.0
allowed (%)	6.0
PDB ID	9lgp

† *R*_meas_ = 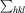
{*N*(*hkl*)/[*N*(*hkl*) − 1]}^1/2^
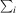
|*I*_*i*_(*hkl*) − 〈*I*(*hkl*)〉|/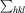

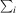
*I*_*i*_(*hkl*).

‡ *R*_p.i.m._ = 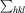
[1/(*n* − 1)]^1/2^
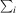
|*I*_*i*_(*hkl*) − *I*(*hkl*)|/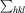

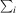
*I*_*i*_(*hkl*).
