# Supplementary material for: Structural insights into the antiviral efficacy of AG7404 against human rhinovirus 3C proteases
Source: IUCrJ. 2026 Jan 1;13(Pt 1):19–30. doi: 10.1107/S2052252525008929 (PMC12809499; doi:10.1107/S2052252525008929)
Supplement: Supplementary file 1 [file m-13-00019-sup1.pdf]

IUCrJ

Volume 13 (2026)

Supporting information for article:

Structural insights into the antiviral efficacy of AG7404 against human rhinovirus 3C proteases

Juyeon Lee, Hye Lim Lee, Hyojin Kim, Yeji Gil, Sang-Ho Lee, Young-Sik Jung, Jin Soo Shin and Inseong Jo

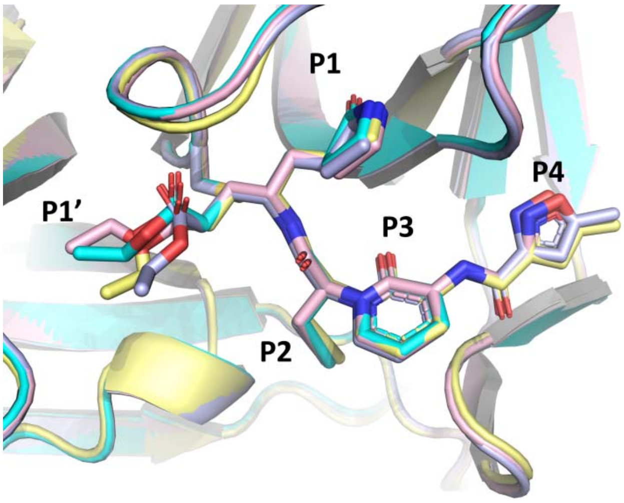

Figure S1 Superposition of four hRV-B14 3C protease protomers, highlighting the AG7404 binding site. A close-up view of the AG7404 inhibitor binding site is shown, with P1', P1, P2, P3, and P4 moieties labeled. Protein chains are colored as follows: chain A in green, chain B in light blue, chain C in pink, and chain D in yellow.

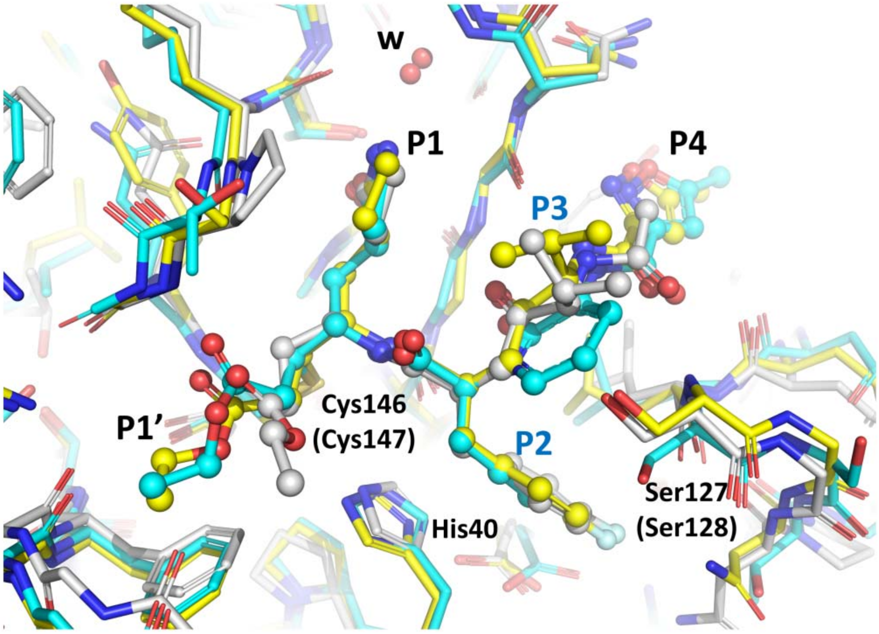

Figure S2 Superposition of active site regions from AG7404-bound hRV-B14 3C protease (cyan), rupintrivir-bound 3C protease from hRV-A2 (yellow; PDB 1CQQ) and hRV-C15 (gray; PDB 6KU8). The AG7404 and rupintrivir molecules are represented with ball-and-stick models.

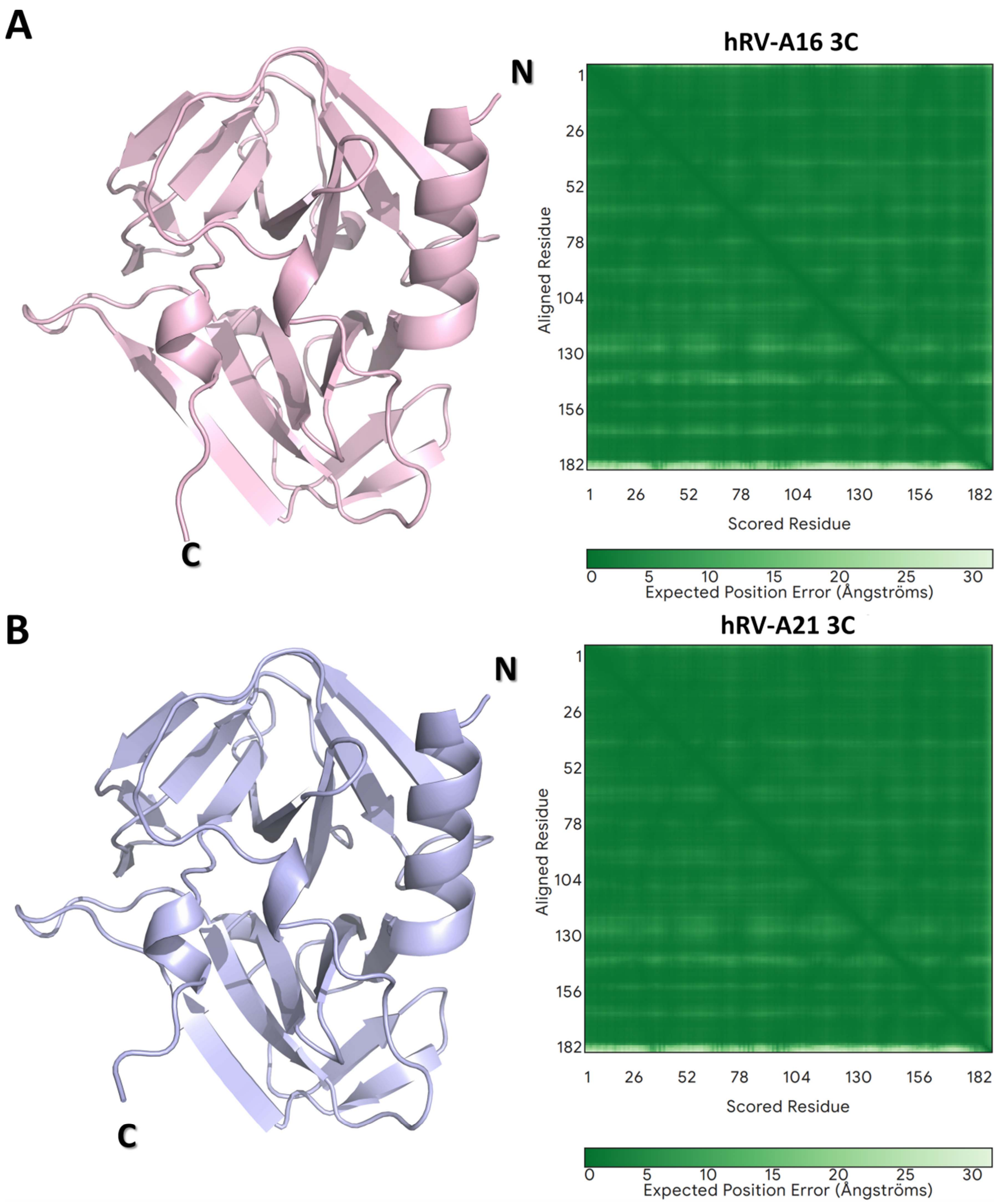

Figure S3 Predicted structures and predicted aligned error (PAE) plots of hRV-A16 and hRV-A21 3C proteases using AlphaFold. The predicted structures of hRV-A16 3C protease (A) and hRV-A21 3C protease (B) are generated by AlphaFold3 and shown as a ribbon diagram in the left panel. The right panel displays the PAE plot, representing the expected positional error (Å) for each residue pair. The green gradient indicates high confidence across the entire structure.

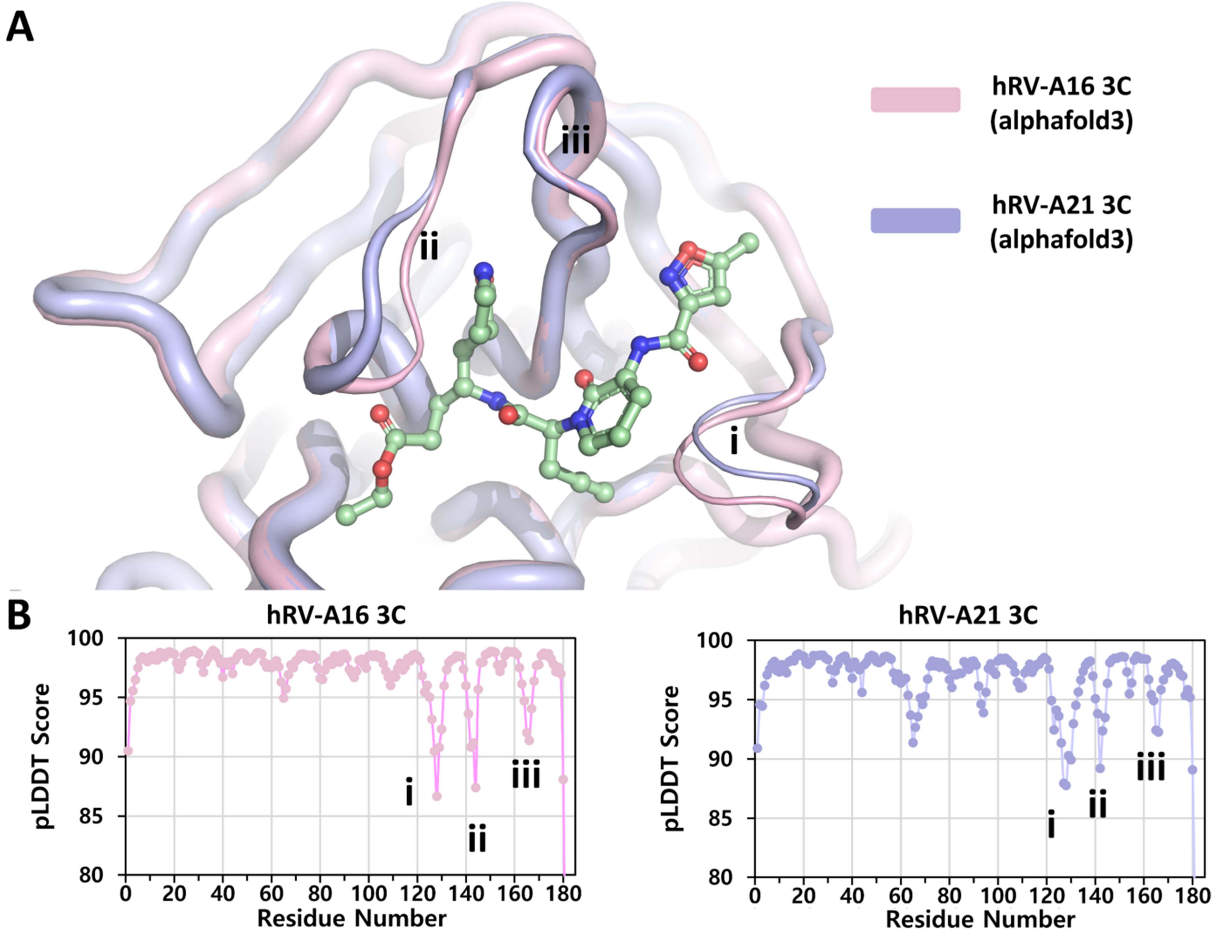

Figure S4 Confidence analysis of the predicted structures of hRV-A16 and -A21 3C proteases.

(A) Superposition of the predicted structures of hRV-A16 (pink) and hRV-A21 (light blue) 3C proteases generated using AlphaFold3. The structures are displayed in tube representation, with the thickness corresponding to the pLDDT score: thicker areas indicate higher confidence, and thinner areas reflect lower confidence. AG7404 (green) is overlaid using the crystal structure of the hRV-B14 3C protease-AG7404 complex. Regions with low pLDDT scores are labeled as i, ii, and iii.

(B) pLDDT score plots for hRV-A16 (left) and hRV-A21 (right) 3C proteases. Residue numbers are plotted on the x-axis, and pLDDT scores are represented on the y-axis. Regions with low pLDDT scores (i, ii, iii) align with those indicated in panel (A).
